# Incidentally exploring natural course of a rare entity: representative case for rosette-forming glioneuronal tumors

**DOI:** 10.1007/s10072-023-06781-1

**Published:** 2023-04-12

**Authors:** Hira Altunbüker, Felix Hinz, Felix Sahm, Stefanie Brehmer, Holger Wenz

**Affiliations:** 1 Department of Neuroradiology, Medical Faculty Mannheim, University Medical Center Mannheim, University of Heidelberg, Theodor-Kutzer-Ufer 1-3, 68167 Mannheim, Germany; 2 Department of Neuropathology, Institute of Pathology, University Hospital Heidelberg, Heidelberg, Germany; 3 Clinical Cooperation Unit Neuropathology, German Consortium for Translational Cancer Research (DKTK), German Cancer Research Center (DKFZ), Heidelberg, Germany; 4 Hopp Children’s Cancer Center Heidelberg (KiTZ), Heidelberg, Germany; 5 Department of Neurosurgery, Medical Faculty Mannheim, University Medical Center Mannheim, University of Heidelberg, Mannheim, Germany

**Keywords:** Rosette-forming glioneuronal tumors, Cerebellar, Entire developmental phase, Resection, Histology

## Abstract

Rosette-forming glioneuronal tumors (RGNT) are extremely rare mostly benign tumors of the central nervous system, which are often studied for its histological aspects despite relatively small numbers of clinical especially radiological knowledge.

Despite the increasing number of publications on different localizations and treatment protocols, the morphologic and temporal development process of this rare tumor entity is not clear. We were able to coincidentally observe the entire course of the tumor growth of a RGNT on subsequent MRI examinations in a typical case with mild clinical symptoms and no other neurological illnesses, thus possible clinical complications were prevented.

## Case

**Fig. 1:**
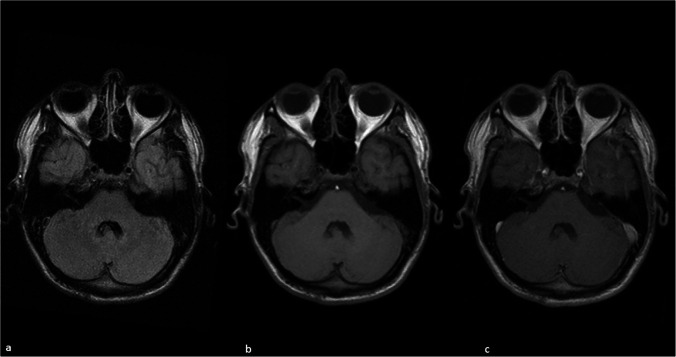
Unremarkable MRI by the time of first appearance (**a**) axial T2 FLAIR imaging (**b**) pre-KM T1 weighted imaging (**c**) post-KM T1 weighted imaging at the level of cerebellopontine peduncle

**Fig. 2:**
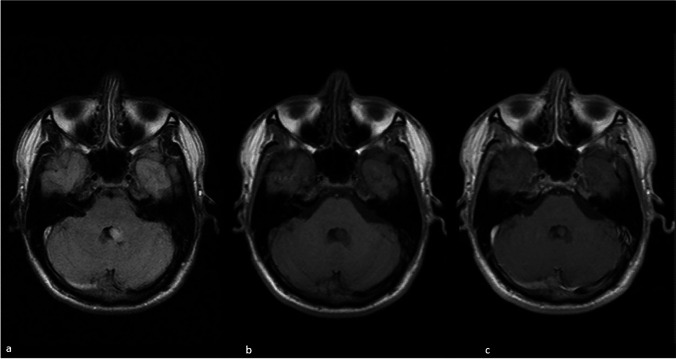
(**a**) Ubiquitous hyperintense signal of the lesion in the axial FLAIR MRI reveals a developing lesion in IV ventricle. (**b**) Axial T1 weighted MRI at the level of cerebellopontine peduncle reveals corresponding hypointense signal of the lesion. (**c**) Apparent ubiquitous T1 relaxation time shortening of the lesion in axial post-gadolinium sequences

An 18-year-old male was admitted to an outpatient clinic with non-specific symptoms such as unease, persistent headache and sleep disturbances. A full examination including head MRI did not indicate any inconspicuous findings (Fig. 1) except an incidental epiphyseal cyst. He declared fluctuating symptoms the following year by the time of routine control of pineal cyst. The control imaging performed externally showed a solitary lesion in the fourth ventricle with gadolinium-enhancement however without reference to obstruction (Fig. 2). Due to the patient’s wishes and mild clinical symptoms, no treatment protocol has been performed and strict clinical follow-up was advised.

**Fig. 3:**
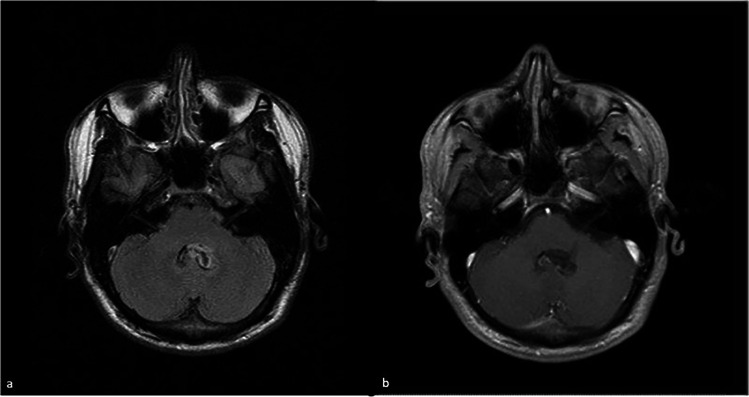
(**a**) Axial FLAIR MRI at the level of cerebellopontine pedincle reveals behavioral change of the lesion with appearance of a cystic component at the center and remanence of the ubiquitous hyperintensity on the edge of cyst. (**b**) Remaining gadolinium-enhancement at the solitary component of the lesion in the axial post-Gadolinium T1 weighted MRI

After 3 years, the patient presented to our institute without any anamnestic changes. However, MRI showed significant progression of the mass, including behavioral change (Fig. 3).

Given the localization of the tumor, microsurgical resection was indicated to avoid possible obstruction and CSF accumulation.

**Fig. 4:**
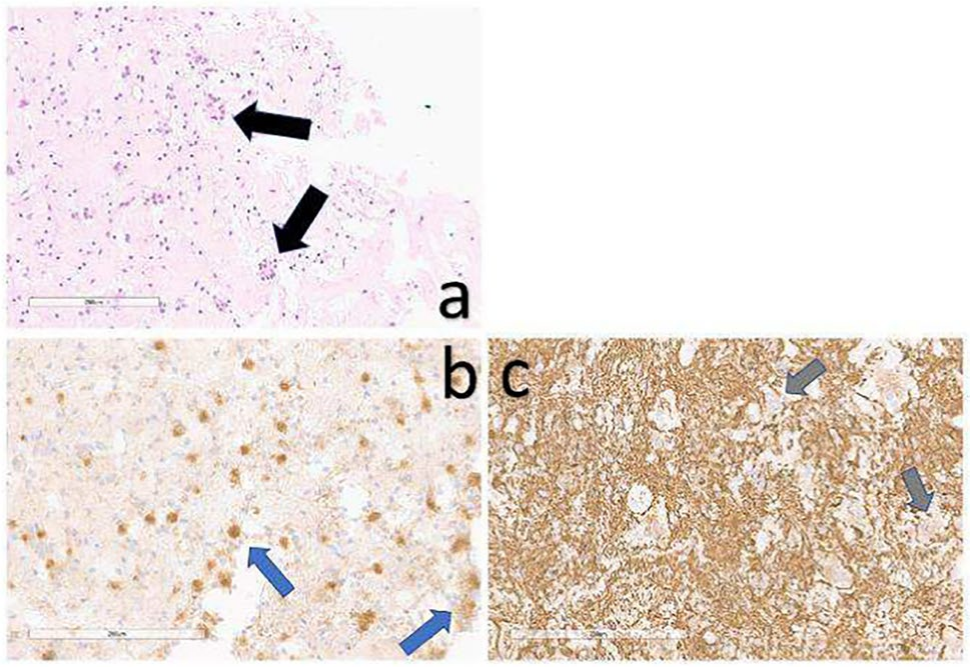
Histology revealed a neuroepithelial tumor with characteristic rosettes containing neuropil (**a**, black arrows). The neuropil stained positive with synaptophysin (**b**, blue arrows). The cells surrounding the neuropil islands expressed no GFAP, indicating the neuronal origin (**c**, grey arrows). In addition, the cells of glial origin were stained positive with GFAP [1]

Histologic findings revealed a rosette-forming tumor with glial and neuronal differentiation (Fig 4a). In immunohistochemistry synaptophysin was positive for rosettes (Fig. 4b), GFAP was positive for glial areas around the rosettes while being negative in the cells around the rosettes (Fig. 4c). In contrast, MAP2 was positive in the cells around the rosettes (Fig. 4d) revealing neuronal origin. In line of the diagnosis NeuN was negative (Fig. 4e). In contrast to ependymomas Olig2 was positive (Fig. 4f).

For validation, the tumor tissue was analysed regarding its epigenetic profile as described before [2]. In this analysis, the tumor sample was successfully characterised as a rosette-forming glioneuronal tumor with a score of 0.99 showing a flat copy number profile confirming morphology.

## Discussion

Rosette-forming glioneuronal tumor (RGNT) is a rare and distinct primary tumor of the nervous system. To our knowledge, this is the first typical case of RGNT in literature in which the entire developmental phase has been observed.

RGNT is a histomorphologic term for this usually benign and relatively slow-growing tumor [1]. Although classified as grade 1 by WHO CNS 5 (2021) [1], there are a growing number of case reports indicating that they may be potentially aggressive [3].

As in our case, these tumors are classically associated with the IV ventricle. However, they may also occur in various other locations, including the lateral ventricle, septum pellucidum, third ventricle, cerebellum, spinal cord, mesiotemporal, midbrain, hypothalamus, hippocampus, pineal gland, visual pathway and even multifocally [3].

Similar to the case we present, the mean age of onset in a meta-analysis by Schlamann et al. is reported to be 27 years (range 6–79) [4]. In addition to headache as the most common symptom, localization-associated symptoms such as hydrocephalus are often found [3].

Gross total resection (GTR) appears to be the first treatment choice; however, progression to subtotal resection (STR) has not yet been demonstrated. Considering the delicate localizations, some cases without surgical option that underwent radio- or chemotherapy have been reported [4].

In this case, we were able to observe for the first time the natural history of RGNT as a rare tumor entity in an exceedingly vivid manner, from onset with mild clinical symptoms not certainly associated with the not significantly space-occupying lesion and no concrete evidence of organic disease (e.g., hydrocephalus), to neoplasia representing a surgical indication for removal.
